# Bis(3-methyl­pyridine-κ*N*)bis­(thio­cyanato-κ*N*)zinc

**DOI:** 10.1107/S1600536811024561

**Published:** 2011-06-30

**Authors:** Jan Boeckmann, Christian Näther

**Affiliations:** a Institut für Anorganische Chemie, Christian-Albrechts-Universität Kiel, Max-Eyth Strasse 2, D-24098 Kiel, Germany

## Abstract

The asymmetric unit of the title compound, [Zn(NCS)_2_(C_6_H_7_N)_2_], consists of one Zn^2+^ cation and two thio­cyanate anions, all situated on special positions with site symmetry .*m*., and one 3-methyl­pyridine ligand. The zinc cation is coordinated by four N atoms of two terminal *N*-bonded thio­cyanate anions and of two symmetry-related 3-methyl­pyridine co-ligands, defining a slightly distorted tetra­hedral coordination polyhedron.

## Related literature

For background to the magnetic properties of Co(II) thio- or seleno­cyanate coordination polymers, see: Boeckmann & Näther (2010 ▶, 2011 ▶); Wöhlert *et al.* (2011 ▶). For isostructural and related compounds with different *N*-donor co-ligands and thio- or seleno­cyanate ligands, see: Bhosekar *et al.* (2010 ▶); Boeckmann *et al.* (2011*a* ▶,*b* ▶,*c* ▶); Taniguchi *et al.* (1987 ▶); Wu (2004 ▶); Zhu *et al.* (2008 ▶).
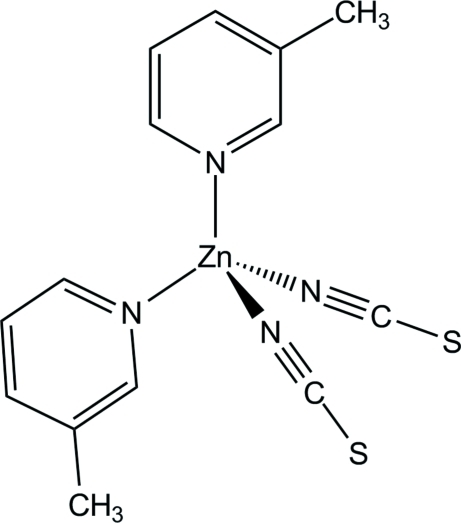


## Experimental

### 

#### Crystal data

[Zn(NCS)_2_(C_6_H_7_N)_2_]
*M* *_r_* = 367.78
Orthorhombic, 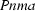

*a* = 8.1510 (4) Å
*b* = 13.7382 (5) Å
*c* = 15.0111 (6) Å
*V* = 1680.94 (12) Å^3^
*Z* = 4
Mo *K*α radiation
μ = 1.71 mm^−1^
*T* = 293 K
0.13 × 0.11 × 0.08 mm

#### Data collection

Stoe IPDS-2 diffractometer
Absorption correction: numerical (*X-SHAPE* and *X-RED32*; Stoe & Cie, 2008) ▶ *T*_min_ = 0.789, *T*_max_ = 0.863
23123 measured reflections
2366 independent reflections
1918 reflections with *I* > 2σ(*I*)
*R*_int_ = 0.048

#### Refinement

*R*[*F* ^2^ > 2σ(*F* ^2^)] = 0.050
*wR*(*F* ^2^) = 0.122
*S* = 1.14
2366 reflections
107 parameters
H-atom parameters constrained
Δρ_max_ = 0.66 e Å^−3^
Δρ_min_ = −0.39 e Å^−3^

### 

Data collection: *X-AREA* (Stoe & Cie, 2008) ▶; cell refinement: *X-AREA* ▶; data reduction: *X-AREA* ▶; program(s) used to solve structure: *SHELXS97* (Sheldrick, 2008 ▶); program(s) used to refine structure: *SHELXL97* (Sheldrick, 2008 ▶); molecular graphics: *XP* in *SHELXTL* (Sheldrick, 2008 ▶) and *DIAMOND* (Brandenburg, 2011 ▶); software used to prepare material for publication: *SHELXL97*.

## Supplementary Material

Crystal structure: contains datablock(s) I, global. DOI: 10.1107/S1600536811024561/wm2500sup1.cif

Structure factors: contains datablock(s) I. DOI: 10.1107/S1600536811024561/wm2500Isup2.hkl

Additional supplementary materials:  crystallographic information; 3D view; checkCIF report

## Figures and Tables

**Table:** 

Zn1—N1	1.928 (4)
Zn1—N2	1.942 (4)
Zn1—N11	2.026 (2)

**Table:** 

N1—Zn1—N2	119.51 (18)
N1—Zn1—N11	108.39 (8)
N2—Zn1—N11	106.32 (9)
N11^i^—Zn1—N11	107.34 (12)

Symmetry code: (i) 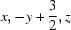.

**Fig. 1.**
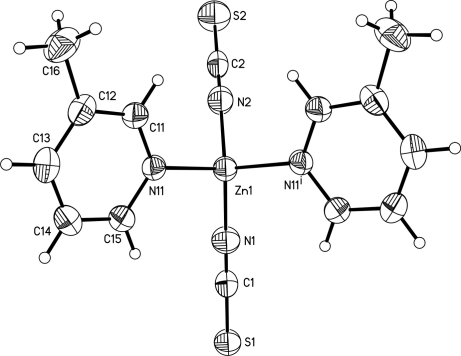
: Molecular structure of the title compound, showing the coordination around Zn2+, with labelling and displacement ellipsoids drawn at the 30% probability level. [Symmetry codes: i = x, -y + 1/2, z.]

**Fig. 2.**
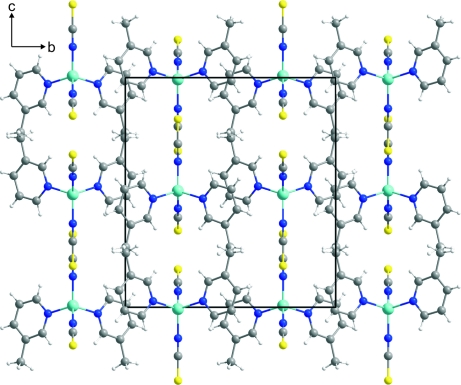
: Packing diagram of the title compound with view along the crystallographic a axis (aqua = zinc; yellow = sulfur; blue = nitrogen; grey = carbon; light-grey = hydrogen).

## supplementary crystallographic information

### Comment

Recently, we have reported about the directed synthesis of one-dimensional and two-dimensional transition metal(II) thio- and selenocyanate coordination polymers with neutral N-donor co-ligands that were obtained by thermal decomposition reactions. The compounds with Co(II) are of special interest because several of them show a slow relaxation of the magnetization which is a rare and very interesting magnetic phenomenon (Boeckmann & Näther, 2010; Boeckmann & Näther, 2011; Wöhlert *et al.*, 2011)). Following this synthetic procedure, powders of low crystallinity are frequently obtained and therefore their structures are difficult to elucidate. Structure determinations of these compounds are of special importance because in the case of coordination polymers containing cobalt(II), both octahedral and tetrahedral coordination polyhedra can occur in these structures. In this context we found out that diamagnetic zinc and cadmium compounds can easily be crystallized in solution and are very often isotypic to their paramagnetic analogues (Bhosekar *et al.*, 2010; Boeckmann *et al.*, 2011a; Boeckmann *et al.*, 2011b; Boeckmann *et al.*, 2011*c*; Taniguchi *et al.*, 1987; Wu, 2004; Zhu, 2008). The structures of the paramagnetic counterparts can then simply be refined applying the Rietveld method. This is the reason why we have determined the crystal structure of the diamagnetic title compound, [bis(thiocyanato-κN)-bis(3-methylpyridine-κN)zinc].

In the crystal structure the zinc cations (site symmetry .*m*.) are bonded to four nitrogen atoms of two terminal thiocyanate anions (site symmetry .*m*.) and to two symmetry-related terminal 3-methylpyridine co-ligands within a slightly distorted tetrahedral coordination polyhedron (Fig. 1 and Tab.1). The discrete complexes are oppositely oriented into columns which spread along the crystallographic *b* axis (Fig. 2). These columns are further arranged in parallel along the crystallographic *a* and *c* axes into a three-dimensional packing.

### Experimental

The title compound was prepared by the reaction of 90.0 mg Zn(NCS)_2_ (0.50 mmol) and 97.3 µ*L* 3-methylpyridine (1.00 mmol) in 1.50 ml water at RT in a closed 3 ml snap cap vial. After three days colourless block like crystals of the title compound were obtained.

### Refinement

All H atoms were discernible in difference maps but were positioned with idealized geometry and were refined isotropically with *U*_eq_(H) = 1.2*U*_eq_(C) for aromatic H atoms and with *U*_eq_(H) = 1.5*U*_eq_(C) for aliphatic H atoms of the parent atom using a riding model with C—H = 0.93 Å (aromatic) and with C—H = 0.96 Å (aliphatic).

### Crystal data

**Table:**

[Zn(NCS)_2_(C_6_H_7_N)_2_]	*F*(000) = 752
*M**_r_* = 367.78	*D*_x_ = 1.453 Mg m^−^^3^
Orthorhombic, *P**n**m**a*	Mo *K*α radiation, λ = 0.71073 Å
Hall symbol: -P 2ac 2n	Cell parameters from 16444 reflections
*a* = 8.1510 (4) Å	θ = 2.0–29.3°
*b* = 13.7382 (5) Å	µ = 1.71 mm^−^^1^
*c* = 15.0111 (6) Å	*T* = 293 K
*V* = 1680.94 (12) Å^3^	Block, colourless
*Z* = 4	0.13 × 0.11 × 0.08 mm

### Data collection

**Table:**

Stoe IPDS-2 diffractometer	2366 independent reflections
Radiation source: fine-focus sealed tube	1918 reflections with *I* > 2σ(*I*)
graphite	*R*_int_ = 0.048
ω scans	θ_max_ = 29.3°, θ_min_ = 2.0°
Absorption correction: numerical (*X-SHAPE* and *X-RED32*; Stoe & Cie, 2008)	*h* = −11→11
*T*_min_ = 0.789, *T*_max_ = 0.863	*k* = −16→18
23123 measured reflections	*l* = −20→20

### Refinement

**Table:**

Refinement on *F*^2^	Primary atom site location: structure-invariant direct methods
Least-squares matrix: full	Secondary atom site location: difference Fourier map
*R*[*F*^2^ > 2σ(*F*^2^)] = 0.050	Hydrogen site location: inferred from neighbouring sites
*wR*(*F*^2^) = 0.122	H-atom parameters constrained
*S* = 1.14	*w* = 1/[σ^2^(*F*_o_^2^) + (0.0581*P*)^2^ + 0.3791*P*] where *P* = (*F*_o_^2^ + 2*F*_c_^2^)/3
2366 reflections	(Δ/σ)_max_ < 0.001
107 parameters	Δρ_max_ = 0.66 e Å^−^^3^
0 restraints	Δρ_min_ = −0.39 e Å^−^^3^

### Special details

**Table:**

Geometry. All esds (except the esd in the dihedral angle between two l.s. planes) are estimated using the full covariance matrix. The cell esds are taken into account individually in the estimation of esds in distances, angles and torsion angles; correlations between esds in cell parameters are only used when they are defined by crystal symmetry. An approximate (isotropic) treatment of cell esds is used for estimating esds involving l.s. planes.
Refinement. Refinement of F^2^ against ALL reflections. The weighted R-factor wR and goodness of fit S are based on F^2^, conventional R-factors R are based on F, with F set to zero for negative F^2^. The threshold expression of F^2^ > 2sigma(F^2^) is used only for calculating R-factors(gt) etc. and is not relevant to the choice of reflections for refinement. R-factors based on F^2^ are statistically about twice as large as those based on F, and R- factors based on ALL data will be even larger.

### Fractional atomic coordinates and isotropic or equivalent isotropic displacement parameters (Å^2^)

**Table:**

	*x*	*y*	*z*	*U*_iso_*/*U*_eq_	
Zn1	0.48061 (6)	0.7500	0.49255 (3)	0.05827 (17)	
N1	0.4523 (5)	0.7500	0.3651 (2)	0.0754 (10)	
C1	0.4513 (5)	0.7500	0.2879 (3)	0.0606 (9)	
S1	0.44866 (18)	0.7500	0.18058 (7)	0.0784 (3)	
N2	0.2888 (5)	0.7500	0.5693 (3)	0.0792 (10)	
C2	0.1682 (5)	0.7500	0.6084 (3)	0.0663 (10)	
S2	−0.00054 (18)	0.7500	0.66536 (11)	0.1021 (5)	
N11	0.6137 (3)	0.63122 (14)	0.52674 (13)	0.0549 (5)	
C11	0.6306 (3)	0.60498 (19)	0.61208 (16)	0.0608 (6)	
H11	0.5791	0.6426	0.6554	0.073*	
C12	0.7202 (4)	0.5254 (2)	0.63937 (19)	0.0662 (7)	
C13	0.7916 (4)	0.4695 (2)	0.5739 (2)	0.0737 (8)	
H13	0.8499	0.4138	0.5892	0.088*	
C14	0.7771 (5)	0.4958 (2)	0.4859 (2)	0.0760 (9)	
H14	0.8267	0.4588	0.4415	0.091*	
C15	0.6884 (4)	0.57729 (19)	0.46463 (17)	0.0642 (7)	
H15	0.6800	0.5957	0.4052	0.077*	
C16	0.7368 (5)	0.5021 (3)	0.7369 (2)	0.0953 (12)	
H16A	0.7898	0.5553	0.7668	0.143*	
H16B	0.8014	0.4441	0.7440	0.143*	
H16C	0.6300	0.4920	0.7622	0.143*	

### Atomic displacement parameters (Å^2^)

**Table:**

	*U*^11^	*U*^22^	*U*^33^	*U*^12^	*U*^13^	*U*^23^
Zn1	0.0690 (3)	0.0527 (2)	0.0531 (2)	0.000	−0.00324 (18)	0.000
N1	0.089 (3)	0.078 (2)	0.0592 (18)	0.000	−0.0136 (17)	0.000
C1	0.066 (2)	0.0520 (18)	0.064 (2)	0.000	−0.0087 (16)	0.000
S1	0.1032 (9)	0.0745 (7)	0.0574 (5)	0.000	−0.0054 (5)	0.000
N2	0.082 (3)	0.063 (2)	0.092 (2)	0.000	0.019 (2)	0.000
C2	0.076 (3)	0.0442 (17)	0.078 (2)	0.000	−0.001 (2)	0.000
S2	0.0739 (8)	0.1173 (12)	0.1150 (12)	0.000	0.0199 (7)	0.000
N11	0.0666 (12)	0.0479 (10)	0.0501 (10)	−0.0036 (9)	−0.0028 (8)	0.0000 (8)
C11	0.0740 (16)	0.0564 (14)	0.0519 (12)	−0.0030 (13)	−0.0015 (11)	0.0003 (10)
C12	0.0725 (17)	0.0607 (14)	0.0655 (14)	−0.0130 (14)	−0.0139 (13)	0.0114 (12)
C13	0.0741 (19)	0.0554 (14)	0.092 (2)	0.0031 (14)	−0.0095 (16)	0.0072 (14)
C14	0.090 (2)	0.0624 (16)	0.075 (2)	0.0102 (15)	0.0062 (15)	−0.0107 (13)
C15	0.0795 (18)	0.0575 (14)	0.0557 (13)	−0.0010 (13)	0.0019 (12)	−0.0029 (11)
C16	0.113 (3)	0.099 (3)	0.074 (2)	−0.004 (2)	−0.020 (2)	0.0275 (17)

### Geometric parameters (Å, °)

**Table:**

Zn1—N1	1.928 (4)	C11—H11	0.9300
Zn1—N2	1.942 (4)	C12—C13	1.377 (5)
Zn1—N11^i^	2.026 (2)	C12—C16	1.505 (4)
Zn1—N11	2.026 (2)	C13—C14	1.374 (4)
N1—C1	1.158 (5)	C13—H13	0.9300
C1—S1	1.611 (4)	C14—C15	1.371 (4)
N2—C2	1.145 (5)	C14—H14	0.9300
C2—S2	1.619 (5)	C15—H15	0.9300
N11—C15	1.338 (3)	C16—H16A	0.9600
N11—C11	1.338 (3)	C16—H16B	0.9600
C11—C12	1.377 (4)	C16—H16C	0.9600
			
N1—Zn1—N2	119.51 (18)	C13—C12—C16	122.6 (3)
N1—Zn1—N11^i^	108.39 (9)	C11—C12—C16	120.4 (3)
N2—Zn1—N11^i^	106.32 (9)	C14—C13—C12	120.2 (3)
N1—Zn1—N11	108.39 (8)	C14—C13—H13	119.9
N2—Zn1—N11	106.32 (9)	C12—C13—H13	119.9
N11^i^—Zn1—N11	107.34 (12)	C15—C14—C13	118.9 (3)
C1—N1—Zn1	173.6 (4)	C15—C14—H14	120.5
N1—C1—S1	179.7 (4)	C13—C14—H14	120.5
C2—N2—Zn1	174.5 (4)	N11—C15—C14	122.0 (3)
N2—C2—S2	179.0 (4)	N11—C15—H15	119.0
C15—N11—C11	118.1 (2)	C14—C15—H15	119.0
C15—N11—Zn1	120.90 (17)	C12—C16—H16A	109.5
C11—N11—Zn1	120.99 (17)	C12—C16—H16B	109.5
N11—C11—C12	123.6 (3)	H16A—C16—H16B	109.5
N11—C11—H11	118.2	C12—C16—H16C	109.5
C12—C11—H11	118.2	H16A—C16—H16C	109.5
C13—C12—C11	117.0 (3)	H16B—C16—H16C	109.5

Symmetry codes: (i) *x*, −*y*+3/2, *z*.
